# Advanced Raman Spectroscopy of Methylammonium Lead Iodide: Development of a Non-destructive Characterisation Methodology

**DOI:** 10.1038/srep35973

**Published:** 2016-10-27

**Authors:** Paul Pistor, Alejandro Ruiz, Andreu Cabot, Victor Izquierdo-Roca

**Affiliations:** 1 IREC - Catalonia Institute for Energy Research, Sant Adrià de Besòs, Spain; 2 ICREA - Institució Catalana de Recerca i Estudis Avançats, Barcelona, Spain

## Abstract

In recent years, there has been an impressively fast technological progress in the development of highly efficient lead halide perovskite solar cells. However, the stability of perovskite films and respective solar cells is still an open point of concern and calls for advanced characterization methods. In this work, we identify appropriate measurement conditions for a meaningful analysis of spin-coated absorber-grade perovskite thin films based on methylammonium (MA) lead iodide (MAPbI_3_) by Raman spectroscopy. The material under investigation and its derivates is the most commonly used for high efficiency devices in the literatures and has yielded working solar cell devices with efficiencies around 10% in our laboratory. We report highly detailed Raman spectra obtained with excitation at 532 nm and 633 nm and their deconvolution taking advantage of the simultaneous fitting of spectra obtained with varying excitation wavelengths. Finally, we propose a fast and contactless methodology based on Raman to probe composition variations and/or degradation of these perovskite thin films and discuss the potential of the presented technique as quality control and degradation monitoring tool in other organic-inorganic perovskite materials and complete solar cell devices.

The success of organic-inorganic lead halides as absorbers in perovskite solar cells in the recent years took the scientific community by surprise. The extremely rapid improvement of solar cell based on CH_3_NH_3_PbI_3_ (methyl ammonium lead iodide, MAPbI_3_) and related compounds lead within few years to efficiencies already surpassing 20% and triggered a lot of research interest from various areas^1^,^2^. There are several fundamental properties that make this material class fascinating and seemingly ideally suited for photovoltaic applications, such as strong direct absorption^3^ and excellent transport properties as a result of very long diffusion lengths and high electron and hole mobilities^4^,^5^,^6^,^7^,^8^. Their easy and low temperature processing^9^, band gap tuneability^10^,^11^ and high open circuit voltages^12^ easily surpassing 1 V make them attractive as a photovoltaic technology and especially suited for tandem applications^13^.

Many fundamental properties of the most widely used hybrid lead halide perovskites are still not fully understood and have to be considered as work in progress. One of the major drawbacks of this technology is that the absorber material itself is relatively unstable. It is known that MAPbI_3_ decomposes easily into PbI_2_ triggered by moisture^14^,^15^ and/or thermal stresses significantly surpassing 100 °C^16^,^17^,^18^. Additionally, the decomposition of the perovskite MAPbI_3_ into PbI_2_ upon illumination^19^,^20^,^21^ has been reported, as well as a theoretical atomistic approach explaining this dominant degradation route^22^. PbI_2_ is therefore commonly found in degraded MAPbI_3_ thin films and is expected to build upon excessive heating/illumination. This material instability not only jeopardizes a successful technological implementation, but also complicates its characterization, as great care has to be taken to measure the pristine material itself and not its decomposition products in all measurements that imply heating and/or exposure to ambient air.

Raman spectroscopy is capable of detecting structural as well as compositional variations on microscopic scale and is easily coupled with photoluminescence studies. It is a versatile technique widely used in conventional thin film applications. Especially in photovoltaic applications it has been established as powerful, non-contact method for the fundamental characterization and quality control^23^,^24^. In thin film photovoltaics, a rigorous control of the optoelectronic properties of the growing layers is of major importance in terms for good reproducibility and homogeneity. We have shown in previous works that advanced Raman spectroscopy can assess relevant properties in photovoltaic materials such as film thickness^24^, crystal structure^25^, defect densities^26^, composition^27^,^28^,^29^ and how it can be used as a versatile and fast tool for quality control and process monitoring^24^.

Hybrid perovskite solar cells have been reported to be especially prone to problems such as inhomogeneity, poor reproducibility and fast degradation. The quality of the absorber layers depends critically on the ambient humidity, temperature, surface treatments of the substrates and seems to vary to a great extent from laboratory to laboratory, often in a not yet well known systematic^30^. These issues call urgently for a reliable way to control the quality and homogeneity of the deposited layers as well as to monitor their degradation.

In this contribution we will investigate the potential of Raman spectroscopy to help systematically in this context. However, up to now there have been only few systematic experimental reports on the vibrational spectra of methyl ammonium lead iodide. The existing literature is controversial on the observable Raman modes for MAPbI_3_ and their position and there is a some mismatch between calculated data^31^,^32^ and experimental attempts^33^,^34^,^35^,^36^,^37^ made so far. In general terms, the experimental data on the vibrational modes of MAPbI_3_ lack detail and resemble very broad bands making the differentiation of specific peaks difficult^33^,^36^. In fact, many studies on the Raman spectra of MAPbI_3_ show features also attributable to the Raman spectrum of PbI_2_^38^, suggesting that severe sample degradation might have taken place before or during the Raman measurements^35^,^37^ as explicitly demonstrated in ref. 21. Only very recently, Grancini *et al.* have published a fundamental work on the vibrational modes of MAPbI_3_ single crystals observing two very broad bands at approximately 110 cm^−1^ and 250 cm^−1 ^39.

Taking into account the relatively low thermal stability of the perovskite absorber, a fast decomposition of MAPbI_3_ under excessive laser light excitation is to be expected and great care has to be taken in order to establish measuring conditions where this is not the case. Here, we present a methodology to measure the highly resolved vibrational modes of spin-coated MAPbI_3_ under the commonly used 532 nm laser excitation and compare it to the near resonant excitation at 633 nm. This includes the derivation of the power density threshold for laser-induced film degradation and a time-resolved study of the MAPbI_3_ decomposition at excessive laser power densities.

For the detailed Raman characterization, bare MAPbI_3_ absorber thin films spin-coated onto FTO-coated glass substrates and sealed in an inert gas atmosphere were used. In order to demonstrate the relevance of the analyzed MAPbI_3_ thin films, solar cells were prepared with these absorber films (with a layer sequence of FTO/TiO_2_/MAPbI_3_/spiro-OMETAD/Ag) and led to working devices with efficiencies between 7–10%, depending on the scan direction. Details on the MAPbI_3_ thin film and solar cell preparation can be found in the experimental methods section and supporting information.

## Results

### Vibrational properties of the methylammonium lead iodine thin films

**Figure 1:**
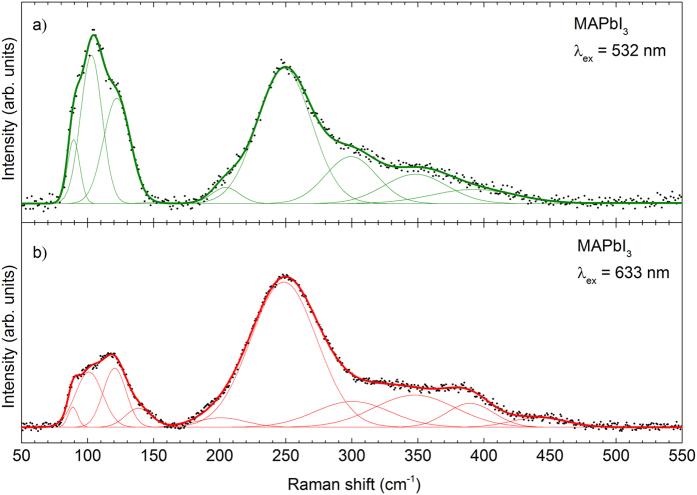
Raman spectra of MAPbI_3_ thin films with excitation at (**a**) 532 nm and (**b**) 633 nm.

Figure 1 shows the Raman spectra of a MAPbI_3_ film after background subtraction (taking into account the baseline and edge filter cut-off as explained in the supporting information) obtained under excitation at 532 nm and 633 nm and keeping the laser power density lower to 26 W/cm^2^ in order to avoid thermal effects on the spectrum. A full power density study has been carried out in order identify the limits when laser-induced sample degradation sets in, which is also presented in the supporting information. Both spectra are characterized by two complex broad structures: one at lower wavenumbers (50–150 cm^−1^) and a second, even broader contribution at higher wavenumbers (175–450 cm^−1^). Table 1 summarizes the peak positions obtained by simultaneous fitting of the spectra during 532 nm and 633 nm excitation with Gaussians, where we followed the methodology developed in refs 40 and 41. Our peak positions can be compared to values reported in the literature for MAPbI_3_ and for the PbI_2_ phase (the main secondary phase expected for this system.) The structure at lower wavenumbers (50–150 cm^−1^) is made up by 4 overlapping contributions (89, 103, 122, and 138 cm^−1^), with a FWHM around to 20 cm^−1^, while the structure with higher wavenumbers (175–450 cm^−1^) is dominated by the peak at 248 cm^−1^ and five less intense contributions (204, 299, 348, 389, and 441 cm^−1^) with a FWHM around 50 cm^−1^.

Theoretical calculations of the vibrational modes of MAPbI_3_ assign lower wavenumber contributions to vibrations of the Pb-I cage, being naturally located in the vicinity of the modes of PbI_2_ (96 cm^−1^, 106 cm^−1^, 113 cm^−1^)^38^. Theoretical calculations of Quarti *et al.* predict librational modes of the organic cations in the 100–200 cm^−1^ range (e.g. at 141 cm^−1^ and 156 cm^−1^)^31^. In consequence, we tentatively assign the mode measured at 138 cm^−1^ to a libration of the organic cation. Quarti *et al.* reported in the experimental part of their work two modes at 250 and 390 cm^−1^, consistent with our analysis and assigned the broad structures at these higher wavenumbers to MA cation torsional modes. The fitting of the experimental data provided by us shows a much higher FWHM of the peaks at the higher wavenumber region (175–450 cm^−1^), in agreement with their more molecular origin. For a highly ordered structure such as the Pb-I cage, a low FWHM is expected. To the contrary, the organic MA molecules have several additional degrees of rotational and torsional freedom and can in this aspect be regarded within the perovskite structure as a disordered arrangement of separated molecules^31^,^42^. This consideration naturally leads to an enhanced dispersion of the vibration frequencies and higher FWHMs. It additionally explains the need for fitting the experimental data with Gaussian curves, (in contrast to a Lorentzian as expected for highly ordered crystal structures). The random-character of this perturbation induced by the organic cation also produces a Gaussian modification of the vibrational energy levels of the Pb-I cage.

The intensity ratio between the Pb-I- and MA-related contributions varies for an excitation at 532 nm as compared to 633 nm. The change of this ratio is attributed to differences in the Raman cross section efficiency of the inorganic and organic contributions under the different wavelengths. Under 532 nm excitation wavelength the energy of the photons (2.3 eV) is close to an energetic band of the MAPbI_3_ at 2.4 eV (at the zone center point), which is attributed to Pb and I atoms by Mosconi in theoretical DFT calculations^42^. This coupling of the 532 nm photons with the 2.4 eV band induces a selective enhancement of the peaks related to vibrations where these atoms are involved, while it should not take place at an excitation with 633 nm (1.9 eV). Similar effects have been reported for different materials such as Cu_2_ZnSnSe_4_^40^, MoS_2_^41^ and are explained in detail for the Zn(S, Se) system^43^.

Comparing our results with the references listed in Table 1, the peak at 250 cm^−1^ identified by us is in agreement with modes reported in refs 31 and 34, and a mode shifted to 280 cm^−1^ in ref. 33. The peaks reported in the other references miss this contribution and mainly coincide with peaks also contributable to the PbI_2_ compound. In order to evaluate the possible impact of the substrate, additional samples were prepared with MAPbI_3_ thin films deposited onto FTO/glass substrates with and without TiO_2_ coating. The analysis gave no evidence of substrate related changes in the Raman spectra (see the supporting information).

### Chemometric evaluation of composed Raman spectra

The curve deconvolution of Raman spectra using Lorentzian and Gaussian curves has been demonstrated to be a powerful technique for the evaluation and quantification of phase evolutions and secondary phases in complex systems. For this evaluation, characteristic peaks for each phase have to be identified and then quantified by an analysis of the relative Raman intensities of the main phase and additional secondary phases^44^,^45^. But this methodology is compromised when the main characteristic peaks of the secondary phases are overlapping with the peaks of the main phase or their concentration is very low. For these cases the use of the selective signal enhancement of certain Raman modes by (near) resonant conditions has been demonstrated as a simple and sensitive methodology that works well even for overlapping signals. However, in cases where the resonant Raman methodology faces problems due to similar bandgaps or strong luminescence inherent to the resonant conditions, a possible alternative is to use a chemometric approach. This approach is widely used in the evaluation of Raman scattering data from organic materials^46^,^47^, and for example in ref. 48.

The Raman signal of the MAPbI_3_ system presents several limitations: intrinsic low signal, strong background, complex broad patterns and strong overlapping of the modes with the possible secondary phases (most prominently PbI_2_). This compromises strongly the detection of secondary phases and the degradation of the MAPbI_3_.

**Figure 2:**
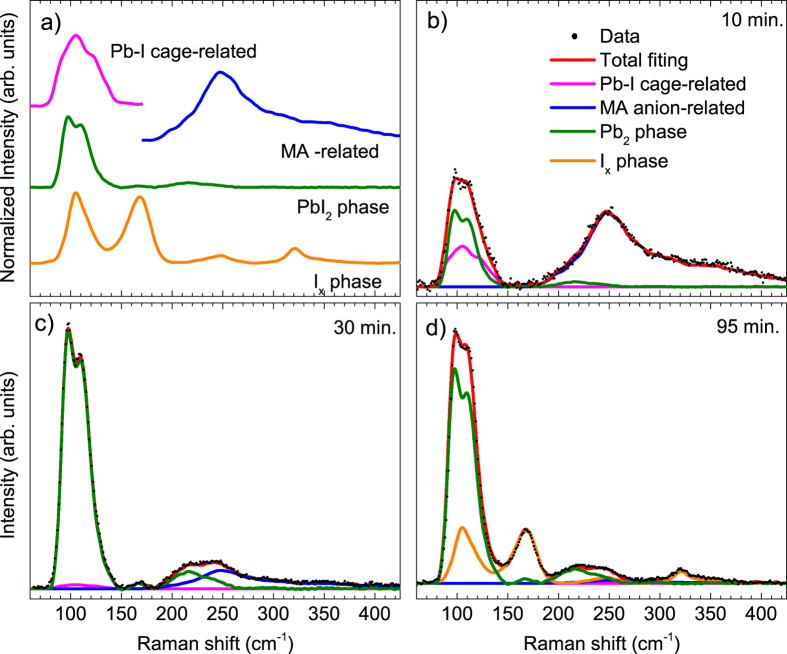
Decomposition of mixed Raman spectra into individual contributions (**a**) Normalised reference Raman spectra from pure compounds used as individual contributions (**b–d)** Mixed Raman spectra and their decomposition into individual contributions obtained after different times of excessive laser exposure of a MAPbI_3_ thin film. These spectra after 10 min, 30 min. and 95 min. are taken from the continuous monitoring of the film degradation displayed in Fig. 3.

The Raman spectra as a function of the wavenumber ***ω*** for mixed compounds can been described as lineal combination of independent signals as described in eq. 1:


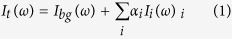


here, ***I***_***t***_ is the total measured Raman signal, ***I***_***bg***_ is the signal of the background, ***I***_***i***_ are the independent signals of the different contributions, and ***α***_***i***_ are the weight factors for each independent contribution which are related to their concentration in the probed volume. The different contributions ***I***_***i***_ shall include all expected phases and are represented by the spectra of the individual pure compounds measured as references. The possibility to decompose the Raman signal into individual terms enables the evaluation and quantification of the different terms: The Raman signal is decomposed directly into independent contributions. To illustrate the methodology, we will give an example. MAPbI_3_ thin films exposed to excessive laser power densities heat up and naturally decompose. As a consequence, during the degradation process different phases may co-exist temporarily with MAPbI_3_ in the probed samples, such as PbI_2_ for example. Furthermore, upon heating, the intensity of the higher wavenumber contribution of MAPbI_3_ (175–450 cm^−1^) associated with MA torsional modes as explained above changes with respect to the lower wavenumber region (75–150 cm^−1^) associated mainly with the Pb-I cage and librational modes of the MA cation. As a consequence, both contributions will be treated as quasi-independent in the following analysis. Figure 2(a) shows different independent contributions identified and taken into account in this work during the heating of MAPbI_3_ thin films with a laser, including the two contributions of MAPbI_3_, a Raman feature coinciding with the PbI_2_ reference and a third phase (I_x_) forming upon prolonged laser exposure. In Fig. 2(b–d) examples are shown of how a mixed Raman spectrum measured during the degradation of the MAPbI_3_ layer can be expressed as a linear superposition of the different terms as described in equation 1.

The possibility to fit the Raman spectra using this methodology enables the evaluation of the presence of different secondary phases (such as the ones shown in this work but not limited to them) that are relevant for an understanding of the impact of different process parameters on the properties of the layers and final devices. Similar analyses of how parameters such as composition, degradation, process temperature, layer thickness, etc. affect the Raman spectra and correlate with optoelectronic properties and device performance have been performed extensively in other photovoltaics material systems such as Si^49^, CIGSSe^24^, CZTSSe^40^,^43^. It has to be stressed, however, that a meaningful interpretation of these correlations always requires a careful calibration of the measured Raman parameter with the optoelectronic properties to be evaluated.

### Continuous assessment of the degradation of MAPbI_3_ thin films under excessive laser exposure

**Figure 3:**
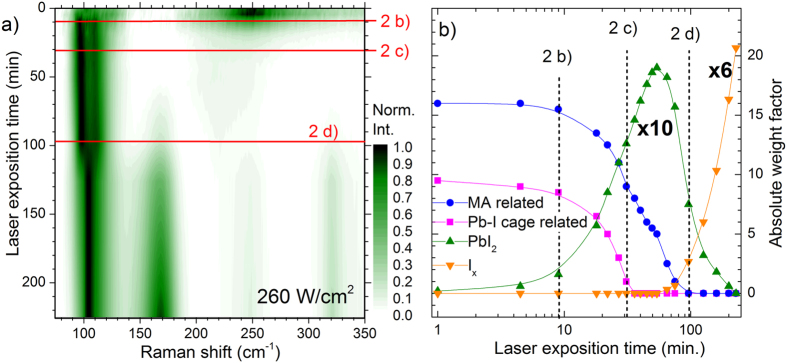
Evolution of the Raman spectra of a MAPbI_3_ thin film upon exposure to laser irradiation with 260 W/cm^2^ and an excitation wavelength of 532 nm. (**a**) Color-coded map of the Raman spectra, where each spectrum has been normalised to its maximum intensity. (**b**) Evolution of the absolute weight factors from the decomposition of the Raman spectra into individual contributions.

In the following we will show how the chemometric methodology introduced above can be used to not only identify sample degradation, but also quantify and monitor it in real time. In our work we have observed how MAPbI_3_ thin films degrade, decompose and desorb if excessive laser power densities are used during Raman analysis. In the supporting information, an illustrative example is presented how a moderate laser power density of 1300 W/cm^2^ (at 532 nm excitation) leads within seconds to the fast transformation of the MAPbI_3_ thin films. The new Raman features coincide with the PbI_2_ reference, which seems to then rapidly desorb from the substrate. Similar effects have been observed by Borchert *et al.* when MAPbI_3_ thin films were monitored during heating by real-time X-ray diffraction^50^. As an example demonstrating the power of the methodology introduced above, we present here the continuous monitoring of Raman spectra of a MAPbI_3_ thin film under the exposure of a moderate laser power density of 260 W/cm^2^. Figure 3 shows the evolution of the Raman spectra with an excitation wavelength of 532 nm as a color-coded map. In this presentation, each row corresponds to one Raman spectra, where the recorded intensity is coded in a color-scale. The ordinate represents the laser exposure (measuring) time. The Raman response of the PbI_2_ compound leads to very intense Raman features and, in consequence, each individual spectrum has been normalized to its maximum in order to distinguish well the different Raman features of all spectra.

The spectra in Fig. 3(a) marked with a red line after 10 min./30 min./95 min. correspond to the spectra presented in the last paragraph in Fig. 2(b–d). The complete transformation of the MAPbI_3_ thin film into a new phase during the first 30 min. of the experiment is observed. As outlined in the introduction, the decomposition of MAPbI_3_ into PbI_2_ upon heating^16^,^18^,^51^ and illumination^19^,^20^,^21^ has been widely reported, and in fact the Raman feature of the newly formed phase coincides with that of PbI_2_. While a further experimental verification of the newly formed degradation products upon laser exposure would be desirable and should be addressed in future work, we here tentatively identify this newly formed compound with PbI_2_. Upon additional laser exposure, the PbI_2_ is further decomposed into a third phase. The origin of this third phase is not yet completely clarified, but the Raman features match peaks reported in literature for polyiodide compounds (I_x_)^52^.

We then decomposed the different Raman spectra measured after increasing laser exposition times into individual spectral contributions as has been outlined in the previous section. The outcome is presented in Fig. 3(b), where the absolute weight factors of the individual contributions are displayed versus the exposure time. We find that during the first 5 minutes, the main contributions stem from the MAPbI_3_. After 5 min, the formation of PbI_2_ starts, being nearly the only contribution after 60 min. After 100 min, the contribution from the PbI_2_ diminishes and gives rise to the third phase which we tentatively have associated with the polyiodide compound.

This example illustrates very well the potential of this methodology to monitor the evolution of different phases with time. While in the presented case the evaluation is only possible in a qualitative manner, in principle a valid calibration taking into account the different Raman sensitivity of the species would also allow for a quantitative determination of the weight portions of the involved phases. The methodology should and will be further refined in this line in the next future. Raman spectroscopy can also be applied to layered structures, obtaining meaningful spectra from buried layers as long as the top layers are transparent to the used laser excitation. This requirement can often be easily met for solar cells by selecting the appropriate excitation wavelength^24^. This means that the analysis can also be performed with finished devices allowing to correlate the Raman results with opto-electronic and performance parameters of the device, enabling a powerful quality and possibly degradation control. We would like to stress here again the possibility to perform these measurements also spatially resolved for inhomogeneity detection.

## Conclusions

In our contribution we have demonstrated the ability of Raman spectroscopy to detect and monitor degradation in perovskite solar cells. We have established non-harming measurement conditions in order to obtain meaningful Raman spectra of MAPbI_3_ thin films at low laser power densities without degrading the film. An excitation at 633 nm was shown to be less harmful than excitation at 532 nm. As a consequence, we were able to report a complete description of the vibrational properties of MAPbI_3_ thin films with high detail comparing spectra measured at an excitation wavelength of 633 nm and 532 nm. Comparison with theoretical calculations in the literature allowed the assignment of the observed bands either to Pb-I bonds or the organic MA molecule. Reference spectra and values taken from the literature revealed similarities but also important differences with respect to the Raman spectrum of PbI_2_, allowing for a clear distinction between the two.

We then suggested a methodology that allows the decomposition of mixed Raman spectra stemming from a mixture of different compounds. Finally, this methodology was tested and applied to the case of a MAPbI_3_ thin film under laser exposure. Here, the decomposition of the film into a phase resembling the Raman features of PbI_2_ within tens of minutes could be monitored. Based on our results, we are very confident that Raman spectroscopy may play an important role as contactless, non-destructive, micro-/macroscale analysis tool for the evaluation of degradation mechanisms in perovskite solar cells in the near future.

## Methods

In order to avoid moisture-induced film degradation, the MAPbI_3_ thin films were spin-casted onto FTO-coated glass substrates inside an argon-filled glovebox. For thin films with a typical thickness of approximately 300 nm, equal molar amounts of MAI and PbI_2_ dissolved in N,N-dimethylformamide (DMF) were used as casting solution. The MAPbI_3_ thin film crystallization was fastened with chlorobenzene as anti-solvent as reported in ref. 53. After spin-casting of the films, these were thermally treated for 6 h at 100 °C within the glovebox to enhance crystallinity in line with optimized conditions found for solar cell performance. Afterwards, the MAPbI_3_ thin film samples were placed into transparent plastic sample boxes which were subsequently evacuated and laminated into plastic foils before taken them out of the glovebox. This way, air exposure could be avoided at all times and the Raman/PL measurements were carried out with the sample sealed in an inert gas atmosphere. Raman spectra were measured with an iHR320 Horiba Jovin Yvon spectrometer coupled to a Raman probe (developed at IREC) in backscattering configuration and macro-spot optics (70 μm spot diameter). All Raman spectra have been calibrated by using a monocrystalline silicon reference and by imposing its main Raman mode at 520 cm^−1^.

## Additional Information

**How to cite this article**: Pistor, P. *et al.* Advanced Raman Spectroscopy of Methylammonium Lead Iodide: Development of a Non-destructive Characterisation Methodology. *Sci. Rep.* **6**, 35973; doi: 10.1038/srep35973 (2016).

**Publisher's note**: Springer Nature remains neutral with regard to jurisdictional claims in published maps and institutional affiliations.

## Supplementary Material

Supplementary Information

## Figures and Tables

**Table 1:** Position of fitted Raman scattering peaks for MAPbI_3_ obtained in this work and comparison with literature data.

MAPbI_3_ Thin film, this work		MAPbI_3_ (Single crystal)	MAPbI_3_ Thin film, literature				PbI_2_ This work	PbI_2_ literature
λ [nm]	Raman peak [cm^−1^]	ref. 39[cm^−1^]	refs 31 and 34[cm^−1^]	ref. 21[cm^−1^]	ref. 33[cm^−1^]	ref. 36[cm^−1^]	Raman peak [cm^−1^]	ref. 38[cm^−1^]
	(*)		62	52				
					69	71		74
*633*	88.8							
			94			94	96.5	96
*532*	102.8							
		109		110	108	108	109.8	106
							117.6	113
*532*	122.1		119					
*633*	138.4					135		
			154			145	166.0	165
					174			
							202.9	205
*532*	204.4							
					215		218.7	220
*633*	248.4	250	250					
					280			
*532*	299.3							
*633*	347.5							
*633*	389.4		390					
*633*	441.4							

The first column lists the excitation that was used for the determination of the specific peak position. (*) At 60 cm^−1^, we observe a maximum in the unprocessed raw data in line with several reports in the literature, but which we assign to an artifact from a combination of Rayleigh decay and laser edge filter as described in more detail in the supporting information.
