# Supplementary material for: Advanced Raman Spectroscopy of Methylammonium Lead Iodide: Development of a Non-destructive Characterisation Methodology
Source: Sci Rep. 2016 Oct 27;6:35973. doi: 10.1038/srep35973 (PMC5081518; doi:10.1038/srep35973)
Supplement: Supplementary Information [file srep35973-s1.pdf]

Supplementary Information:

Advanced Raman Spectroscopy of Methylammonium Lead Iodide: Development of a Non-destructive Characterisation Methodology

Paul Pistor,[,1] Alejandro Ruiz,[1] Andreu Cabot,[1,2] Victor Izquierdo-Roca[1]*

1 IREC - Catalonia Institute for Energy Research, Sant Adria de Besos, Spain
2 ICREA -Institució Catalana de Recerca i Estudis Avançats, Barcelona, Spain

Corresponding Author: * Paul Pistor, eMail: ppistor@irec.cat

Summary: In this supporting information, we show details of the background subtraction process for the measured raw Raman data accompanied by additional Raman reference spectra of PbI_2 powders and photoluminescence measurements of the $MAPbI_3$ thin films. The importance of measuring the Raman spectra at low power densities will be proven by a detailed power density study and the correct non-destructive measurement conditions will be presented. In addition, details of the $MAPbI_3$ sample preparation will be enclosed, followed by a description of the solar cell device processing. Finally, first IV measurements of solar cell devices under simulated AM1.5 illumination are shown for different measurement conditions with a short discussion of the observed hysteresis effects.

Raman spectroscopy: Background subtraction and additional Raman data

In Figure S1 and its caption we explain how the background and its contributions are subtracted from the raw data. Figure S2 shows the Raman spectra of a $MAPbI_3$ thin film in comparison with PbI_2 reference powder. The inset shows the photoluminescence (PL) spectrum of the investigated $MAPbI_3$ thin films with a PL maximum at 1.62 eV

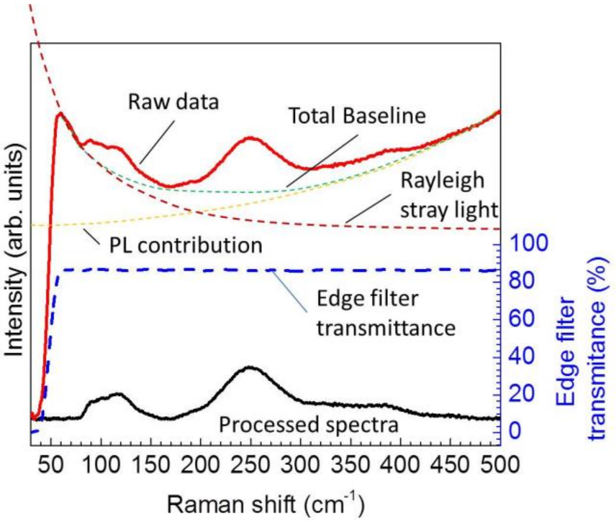

Figure S1. Background subtraction from Raman spectra. The red solid line (above) represents the raw measured data of a $MAPbI_3$ thin film with excitation at 633 nm (6.5 W/cm^2), including the background. The background originates towards lower wave numbers from the reflected laser signal (red dotted line) and towards higher wave numbers from the increasing photoluminescence signal (yellow dotted line). Below, the experimental Raman data after background subtraction (black) as well as the transmittance of the applied edge filter are shown. .

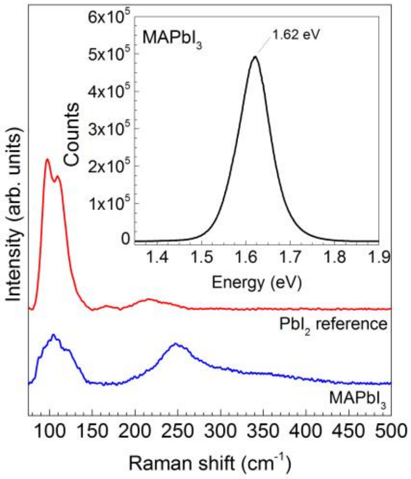

Figure S2. Raman spectra of the $MAPbI_3$ thin film in comparison with a PbI_2 powder reference. Inset: Photoluminescence spectra of $MAPbI_3$ thin film.

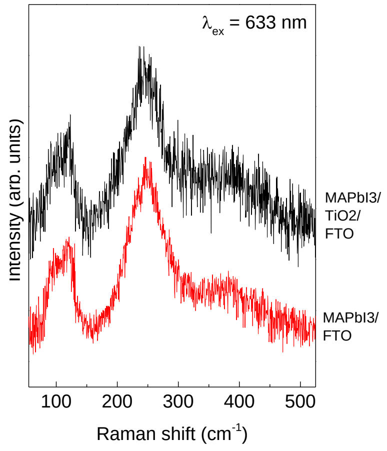

Figure S3. Comparison of the Raman spectra of $MAPbI_3$ thin films deposited on FTO/glass substrates with and without TiO_2 coating.

Identification of non-destructive Raman measurement conditions

In order to clarify the impact on the $MAPbI_3$ thin films and to identify the laser power density threshold for which sample degradation sets in, a detailed power density study has been performed for the 532 nm and 633 nm excitation wavelength. Figure S4 shows the absolute Raman spectra for the 532 nm excitation and power densities between 2.6 W/cm^2 and 1.3 kW/cm^2. No changes in the Raman spectra are observed for power densities up to 26 W/cm^2. Increasing the power density to 260 W/cm^2 leads to an intensity reduction of the contribution at 100-150 cm^{-1} mainly related to the Pb-I cage while the MA-related contribution around 250 cm^{-1} does not experiment any significant changes. This suggests that, in this power density range, the perovskite structure experiments degradation by a breaking of the Pb-I bounds, while the organic contributions seem unaffected. For powers density higher than 26 W/cm^2, the peak intensity variation and appearance of new peak contributions evidence film degradation and the formation of PbI_2. Using a power density of 1300 W/cm^2, for example, the $MAPbI_3$ is converted within seconds into PbI_2, which thereafter is desorbed from the substrate, as is shown in figure S5.

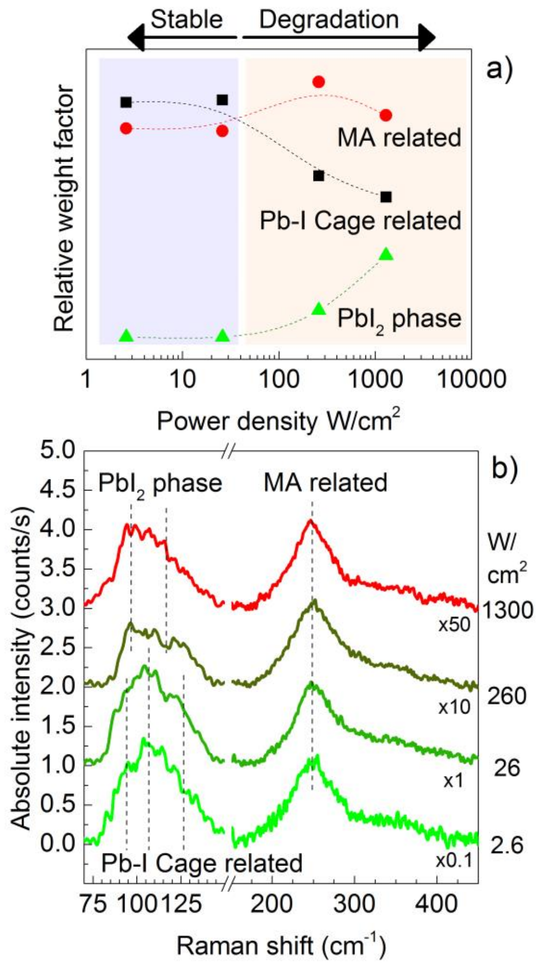

Figure S4. Impact of laser excitation power (at 532 nm) on the Raman spectra of $MAPbI_3$ thin films. Alterations of the Raman spectra above a density threshold (> 26 W/cm^2) indicate a laser-induced sample modification. a) Relative weight factors (the weight factor of the specie divided by the sum of the weight factors of all species) as derived for the chemometric data interpretation in the main manuscript. b) Absolute Raman spectra.

For the case of the excitation at 633 nm, no modifications of the spectra were found for the studied power density range up to 260 W/cm^2, which was the upper limit for our experimental setup. This lower sensibility to degradation has been attributed to the reduced optical absorption coefficient of the $MAPbI_3$ at this photon energy, (from 10^5 cm^{-1} (532 nnm) to $3x10^4$ cm^{-1} (633 nm)).

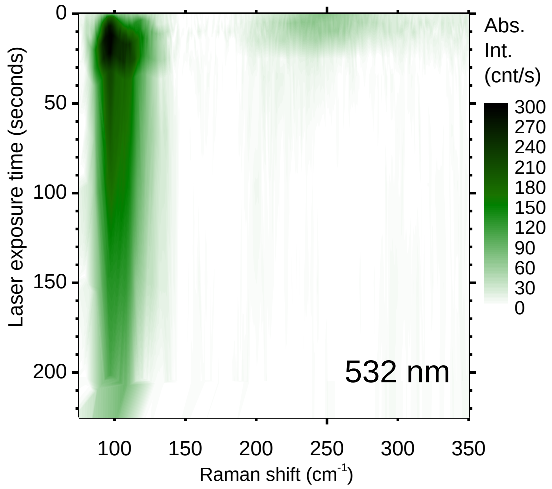

Figure S5. Series of Raman spectra at an excessive laser power density of 1300 W/cm^2 and an excitation wavelength of 532 nm. The $MAPbI_3$ thin film is decomposed into PbI_2 within seconds and subsequently desorbed from the substrate.

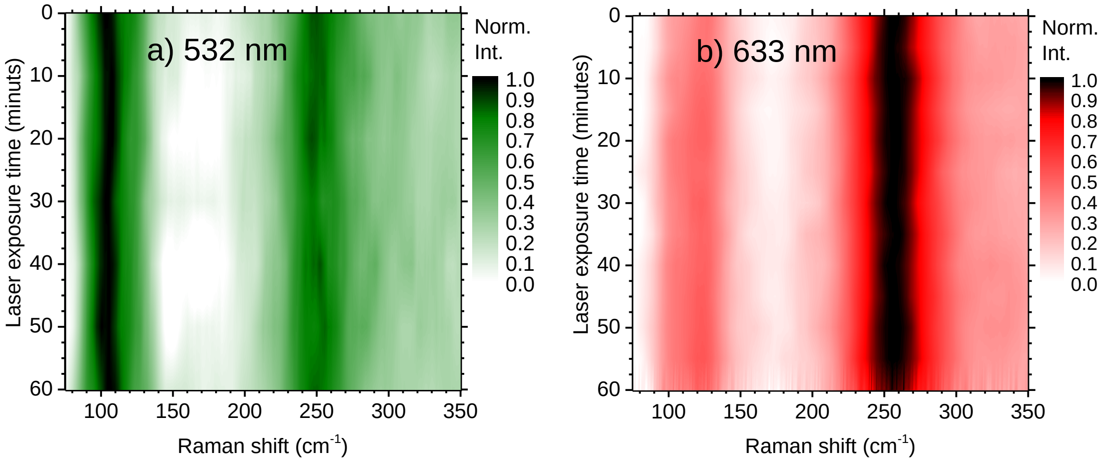

Figure S6. Series of Raman spectra within the stable power density range verifying no change in the Raman spectra with time. Measurements were performed with 5 minutes integration time and a) 532 nm, 26 W/cm^2 and b) 633 nm, 260 W/cm^2.

In order to confirm the absence of any laser-induced degradation below the power density threshold, a study of the evolution of the Raman spectra with laser exposition time has been performed (Figure S6). Within one hour of laser exposure no change in the Raman features could be observed for excitation with a) 532 nm, 26 W/cm^2 and b) 633 nm, 260 W/cm^2.

Experimental details of the $MAPbI_3$ thin film preparation.

For the casting solution, equal molar amounts of MAI and PbI_2 were dissolved in N,N-dimethylformamide (DMF) (45 weight %). The casting solution was dropped onto the substrate and spinned at 5000 rpm for 30s. After 5s spinning time the chlorobenzene anti-solvent was added to the spinning substrate to fasten the crystallisation. With adding of the anti-solvent, the samples immediately turned dark brown. The crystallisation of the $MAPbI_3$ was enhanced by drying the films for 6 h on a hot plate (100ºC) inside the glove box.

Experimental details of the solar cell preparation.

Solar cells were fabricated in the commonly used superstrate configuration and a glass/FTO/TiO_2/$MAPbI_3$/spiro-OMETAD/Ag stack. First, a blocking TiO_2 layer was spin-coated onto the FTO and subsequently annealed in air (30 min., 500ºC), followed by a mesoporous TiO_2 layer (5 vol. % TiO_2 paste (Dyesol) in ethanol, 30 min. 500ºC annealing). $MAPbI_3$ was spin-coated on top as described above and the samples heated inside the glovebox on a hot plate for 6h (100ºC). The spiro-OMETAD hole-conducting layer and silver contact layer were subsequently spin-casted and thermally evaporated on top.

Solar cell characterization

IV-curves of the solar cells were measured at a temperature of 25ºC in the dark and under a simulated AM1.5 illumination (100mW/cm^2). Figure S7 shows the illuminated IV-curve of a representative solar cell in different measurement conditions: in forward direction (from short-circuit towards open circuit conditions) and starting from different voltages for the reverse voltage sweeps (from open circuit to short-circuit conditions). A significant influence of the measurement conditions on the IV-characteristics has been observed. As a consequence, it is clear that the extracted solar cell parameters as displayed in Table S1 for these devices are not stabilized. This clearly invites for optimization: A more detailed IV-characterization and optimization of our devices is currently under investigation and results are only presented here to show the relevance of the investigated material.

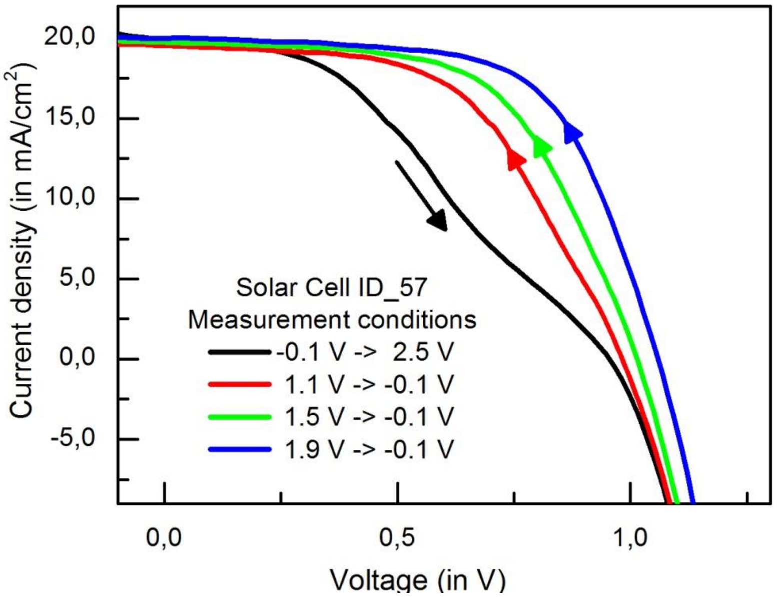

Figure S7: IV-curves under simulated AM1.5 illumination of a glass/FTO/TiO_2/$MAPbI_3$/spiro-OMeTAD/Ag device for different starting points of the IV-measurements.

Table S1: Solar cell parameters of the device presented in Figure S5, extracted from the IV-curves under the different measurement conditions.

Measurement conditions	j_{SC} (mA/cm^2)	FF (%)	V_{OC} (mV)	Efficiency (%)
-0.1 V -> 2.5 V	20.0	37.7	943	7.1
1.1 V -> -0.1 V	19.5	54.8	980	10.5
1.5 V -> -0.1 V	19.7	58.9	1013	11.8
1.9 V -> -0.1 V	20.0	63.4	1058	13.4
